# TRAIL and DcR1 Expressions Are Differentially Regulated in the Pancreatic Islets of STZ- versus CY-Applied NOD Mice

**DOI:** 10.1155/2011/625813

**Published:** 2011-11-28

**Authors:** Ercument Dirice, Sevim Kahraman, Gulsum Ozlem Elpek, Cigdem Aydin, Mustafa Kemal Balci, Abdulkadir Omer, Salih Sanlioglu, Ahter Dilsad Sanlioglu

**Affiliations:** ^1^Section of Islet Cell and Regenerative Medicine, Joslin Diabetes Center, Harvard Medical School, Boston, MA 02215, USA; ^2^Human Gene and Cell Therapy Center of Akdeniz University Hospitals and Clinics, 07058 Antalya, Turkey; ^3^Department of Medical Biology and Genetics, Faculty of Medicine, Akdeniz University, 07058 Antalya, Turkey; ^4^Department of Pathology, Faculty of Medicine, Akdeniz University, 07058 Antalya, Turkey; ^5^Division of Endocrinology and Metabolic Diseases, Faculty of Medicine, Akdeniz University, 07058 Antalya, Turkey; ^6^Division of Endocrinology and Diabetes, Department of Medicine, University of Massachusetts, Worcester, MA 01545, USA

## Abstract

TNF-related apoptosis-inducing ligand (TRAIL) is an important component of the immune system. Although it is well acknowledged that it also has an important role in Type 1 Diabetes (T1D) development, this presumed role has not yet been clearly revealed. Streptozotocin (STZ) and Cyclophosphamide (CY) are frequently used agents for establishment or acceleration of T1D disease in experimental models, including the non-obese diabetic (NOD) mice. Although such disease models are very suitable for diabetes research, different expression patterns for various T1D-related molecules may be expected, depending on the action mechanism of the applied agent. We accelerated diabetes in female NOD mice using STZ or CY and analyzed the expression profiles of TRAIL ligand and receptors throughout disease development. TRAIL ligand expression followed a completely different pattern in STZ- versus CY-accelerated disease, displaying a prominent increase in the former, while appearing at reduced levels in the latter. Decoy receptor 1 (DcR1) expression also increased significantly in the pancreatic islets in STZ-induced disease. Specific increases observed in TRAIL ligand and DcR1 expressions may be part of a defensive strategy of the beta islets against the infiltrating leukocytes, while the immune-suppressive agent CY may partly hold down this defense, contributing further to diabetes development.

## 1. Introduction

Type 1 diabetes (T1D) results from selective autoimmune destruction of the pancreatic beta islets by the infiltrating inflammatory cells [1, 2]. Among the various molecules known to take role in the disease course, the recently defined Tumor-necrosis factor- (TNF-) related apoptosis-inducing ligand (TRAIL) holds a unique position. There is evidence for both destructive and especially protective roles for TRAIL in T1D unlike other TNF-alpha family members, which are mainly known for destructive effects on pancreatic beta cells [3–7]. TRAIL is a type 2 membrane protein that can interact with four different membrane receptors and a soluble osteoprotegerin receptor in humans. Of the transmembrane receptors, TRAIL-R1 (DR4) and TRAIL-R2 (DR5) serve as death receptors, while they can also activate nuclear factor NF-*κ*B pathways [8–10]. TRAIL-R3 (DcR1) and TRAIL-R4 (DcR2) are called decoy receptors that are not capable of triggering apoptosis, yet may activate NF-*κ*B [11]. In mice, on the other hand, only three membrane TRAIL receptors have been defined, one of which is a death receptor and shows great homology with the human DR5 [10]. The other two are decoy receptors with no intracellular domains. 

The exact role of TRAIL and its receptors in the development of T1D is yet to be identified. Blocking of the TRAIL function with a soluble TRAIL receptor was shown to accelerate diabetes in a study conducted on NOD mice [3]. It was also presented in the same study that TRAIL-deficient C57BL/6 mice displayed an earlier onset of diabetes following application of multiple low doses of streptozotocin (STZ) compared to normal mice. Results from these two experimental patterns along with a few others suggested a protective role for TRAIL against autoimmune inflammation. On the other hand, presence of TRAIL was reported as a part of the T-cell response repertoire in beta cell destruction [4]. In the mentioned study, activated T-cell lines derived from 29 children with new-onset T1D displayed elevated TRAIL expression in the infiltrating T cells. Our group has also reported correlation between high TRAIL expression levels and increased beta cell death in human pancreas [12, 13]. Thus, there are also reports contrarily suggesting a destructive role for TRAIL in diabetes development. Clarification of this seemingly controversial role of TRAIL is necessary for a thorough understanding of the disease mechanism. 

Nonobese diabetic (NOD) mice constitute very suitable *in vivo* models for studies in human T1D. An important portion of what is known today about this disease has been provided by investigations using NOD mice. This is because studying with human pancreatic tissue is highly constricted due to both medical and ethical issues and also because NOD mice represent the closest T1D model to human disease [14]. Although NOD mice develop spontaneous diabetes, investigators frequently use diabetes-accelerating agents for usually a quicker presentation of disease and a higher prevalence [5, 15]. Streptozotocin (STZ) and cyclophosphamide (CY) are two well-known and widely used agents for such purpose. STZ is taken into cells via GLUT2 receptors due to its resemblance to glucose molecule, which limits its action primarily to the beta cells. Its toxic effect is exerted through mechanisms such as nitric oxide formation, alkylation, and DNA fragmentation [16–18]. CY, on the other hand, is thought to directly affect (suppress) the CD4^+^ CD25^+^ T reg cells, easing development of diabetes [19]. 

It is thus well accepted that while the induction/acceleration of diabetes provides suitable disease models, different diabetes-inducing agents usually have distinct disease-causing mechanisms. This difference may well be reflected as differential effects on the elements of the immune system such as the TRAIL ligand. In this study, we hypothesized that expression levels of TRAIL ligand and its transmembrane receptors may be distinctly influenced by STZ and CY. We used the diabetes-prone NOD mice to test our hypothesis. Insulitis-resistant diabetes-free nonobese diabetes resistant (NOR) mice were used as controls, and went through the same experimental procedures as the NOD mice. We found that TRAIL ligand expression levels were elevated in the pancreatic islets following STZ injection in NOD mice. In contrast, a prominent reduction was observed following induction with CY. DcR1 expression increased along with TRAIL ligand in the islets in STZ-accelerated diabetes, while no significant change was observed following CY application. These results indicate differential effects of two different diabetes-inducing agents on the expression profiles of TRAIL ligand and its DcR1 receptor, while providing implications on their roles in T1D development.

## 2. Materials and Methods

### 2.1. NOD and NOR Mice

Female NOD/LtJ (NOD) and NOR/LtJ (NOR) mice were purchased from Jackson Laboratory (Bar Harbor, Me, USA), which reached our lab at 5 weeks of age. All mice were housed at an isolated section in the Experimental Animal Care and Production Unit of Akdeniz University, Faculty of Medicine, with a 12 h light-dark cycle. The mice were quarantined at the mentioned unit for three weeks before application of any experimental procedures. All animal work was conducted under the approval of the Institutional Animal Care and Use Committee of Akdeniz University and in accordance with the Helsinki Declaration guidelines. NOR mice were used to set proper control groups for the procedures held in NOD mice and put through exactly the same protocols. 

### 2.2. Induction and Evaluation of Diabetes

 Streptozotocin (STZ) (Sigma-Aldrich) was prepared in citrate buffer solution immediately before i.p. administration to provide the appropriate pH (4.2–4.5) for its activation. The cyclophosphamide (CY) (Endoxan) solution was also prepared immediately before the i.p. injection, in 0.9% saline. A single dose of STZ (150 mg/kg) or CY (200 mg/kg) was administered to prediabetic (10 weeks old) female NOD mice and also to age-matched female NOR mice. Thus, all the mice included in this study received one of the mentioned diabetes-accelerating agents or vehicle. Mice were tested for onset of diabetes through measurements of nonfasting blood sugar once every other day using blood collected from the tail vein with a portable glucose meter (Accu-Check Go; Roche, Nutley, NJ, USA). Mice with a blood sugar level of 250 mg/dL or over on two consecutive measurements were considered diabetic.

### 2.3. Dissection of Pancreata

Pancreata were carefully isolated from the related organs and tissues in mice under ketamine-xylazine anesthesia and placed in 10% formalin. Mice were immediately sacrificed afterwards. The sample collection times after injections were as follows: days 2, 4, 7, 11, and 14 from STZ-applied NOD and NOR mice and days 1, 4, 7, 14, and 21 for CY-applied NOD and NOR mice. Pancreatic samples from three mice were isolated at each point for NOD mice. Two samples per point were evaluated for NOR mice for CY application. Tissues collected from STZ-applied NOR mice were not evaluated as control samples, as stated below, as these mice were very severely affected by STZ induction. Pancreatic tissues were also isolated at day 0 (before agent injection) from at least 3 other NOD and NOR mice.

### 2.4. Immunohistochemistry

The formalin-fixated pancreatic tissues were paraffin embedded, and serial sections (4-5 *μ*m) were obtained from different levels of the blocks. Haematoxylin-eosin staining was used for general structural observation. Immunostaining was performed on formalin-fixed, paraffin- wax embedded sections. Expression levels of the TRAIL ligand and its receptors were detected with suitable rabbit anti-mouse primary antibodies (polyclonal, diluted 1 : 100, Prosci-Inc, Calif, USA). The brief procedure is as follows: sections from each block were deparaffinized and heated in a microwave oven for 10 min for antigen retrieval. Endogenous peroxidase was blocked using 3% hydrogen peroxide in methanol for 10 min. Each incubation step was followed by thorough washing of the slides in distilled water and phosphate-buffered saline (PBS) (0.001%, Sigma). After incubation with primary antibodies for 60 min, sections were allowed to react with the secondary biotinylated antibody (Lab Vision/Thermo Fisher Scientific) for 15 min and streptavidin peroxidase (TS-125-HR; Lab Vision) for 15 min. Finally, all slides were treated with DAB reagent (Lab Vision/Thermo Fisher Scientific) for equal time intervals across all samples for color development and counterstained with Mayer's haematoxylin. In all series, relevant positive controls included sections from lymph nodes. Tissue samples that were stained with the secondary antibody alone were used as negative controls.

### 2.5. Immunohistochemical Scoring

A pathologist (G. O. Elpek) who had no prior knowledge about the tissue origins interpreted the slides for both distribution (percentage of the positively stained cells) and intensity of immunostaining. In each case, positive and negative cells were counted at 400x magnification and the percentage was calculated as the number of positive cells relative to the total number of cells counted. Intensity of staining was classified as (0) negative, (1) weak, (2) moderate, and (3) strong for positivity in nuclei and/or cytoplasm. Marker distribution was scored as (0) less than 10%, (1) between 10% and 40%, (2) between 40% and 70%, and (3) for more than 70% of the cells stained positive. The final staining score was determined as the total of the intensity and marker distribution scores. The number of islets evaluated ranged from 4 to 12 (mean: 8,3 ± 3,7) for the assessment of immunostaining in endocrine cells. Insulitis-free areas were used for evaluation in the islets surrounded with infiltrating lymphocytes.

### 2.6. Statistical Analysis

 Statistical analysis was performed with SPSS 13.0 for Windows (SPSS Inc. Chicago, Ill, USA). Normality tests were carried out in the groups by Shapiro-Wilk test. Statistical evaluation continued with Mann-Whitney *U* test which allows comparison of two independent groups, for evaluation of the statistical significance of the differences observed. Statistical significance was considered at 5% probability level (*P* < 0.05).

## 3. Results

### 3.1. Diabetes Development Was Differentially Accelerated in NOD Mice by Single Injection of Streptozotocin (STZ) or Cyclophosphamide (CY) at 10 Weeks of Age

**Figure 1:**
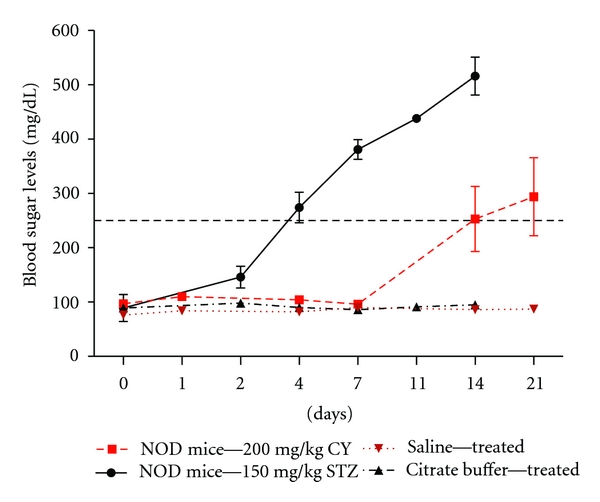
Mean blood glucose levels following STZ- or CY-inductions. NOD mice received single intraperitoneal injections of 150 mg/kg STZ or 200 mg/kg CY or vehicle (physiological saline or citrate buffer) at 10 weeks of age. Fifteen mice were used for initial inductions in both STZ and CY groups. Three mice were sacrificed at each point for immunohistochemical pancreas analysis starting from day 1 for the CY group and day 2 for the STZ group. The given values represent the mean blood glucose values for the live subjects, including those to be sacrificed, at each point.

Various different application doses of STZ have been reported in studies conducted on NOD mice applied at varying ages [20, 21]. We applied 200 mg/kg STZ as a starter dose in a small number of animals (*n* = 5) as a pilot study. The mentioned dose displayed a very fast and lethal effect on the mice, where 3 out of 5 mice died at day 6 of application. We then applied a decreased dose of 150 mg/kg STZ, which did not display undesired immediate toxic effects. A total of 15 female NOD mice at 10 weeks of age were then injected with 150 mg/kg STZ. Some of the mice displayed symptoms of the disease starting at day 4, such as blood sugar levels of over 250 mg/dL and progressive weight loss. The mean blood sugar levels measured in NOD mice after day 4 were over 250 mg/dL at all points (Figure 1). On the other hand, CY was applied at a single dose of 200 mg/kg to 15 female NOD mice at 10 weeks of age. These mice developed T1D at a slower course and at a lower incidence compared to the STZ-induced animals (Figure 1). Diabetic blood sugar levels were reached starting from day 11. The proportions of the diabetic subjects in the sacrificed groups at each point (*n* = 3) for marker evaluation in the 150 mg/kg STZ-applied and 200 mg/kg CY-applied experimental groups are presented in Table 1. 

### 3.2. TRAIL Ligand Expression Levels Followed a Completely Different Pattern in the Pancreatic Islets of STZ-versus CY-Applied Animals

**Figure 2:**
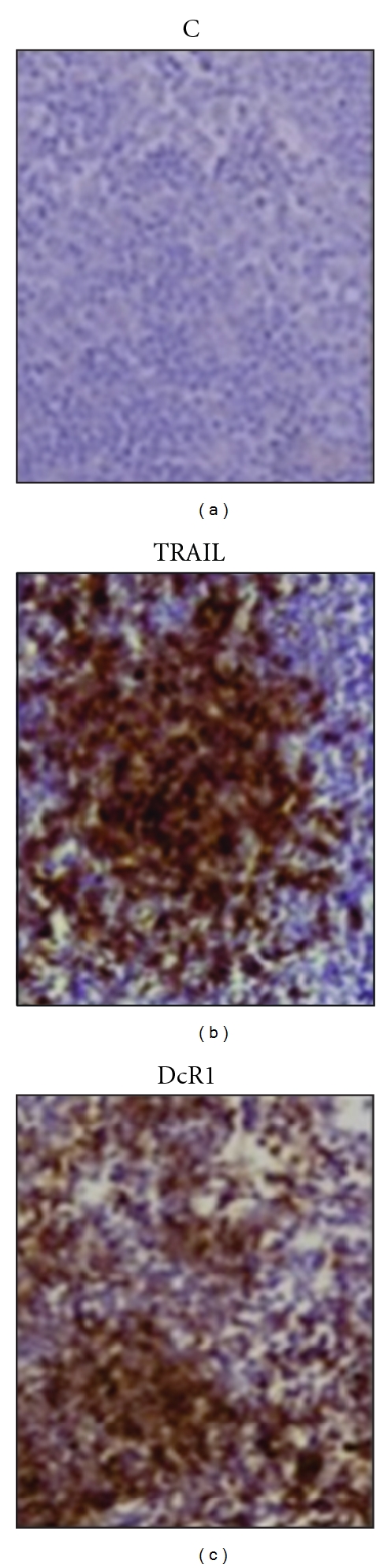
Lymph node staining of the TRAIL ligand and its DcR1 decoy receptor. Specificities of the primary antibodies were confirmed on lymph node sections. Stainings for TRAIL and DcR1 are shown. C stands for the negative control staining, where only the secondary antibody was used in the immunohistochemical procedure.

**Figure 3:**
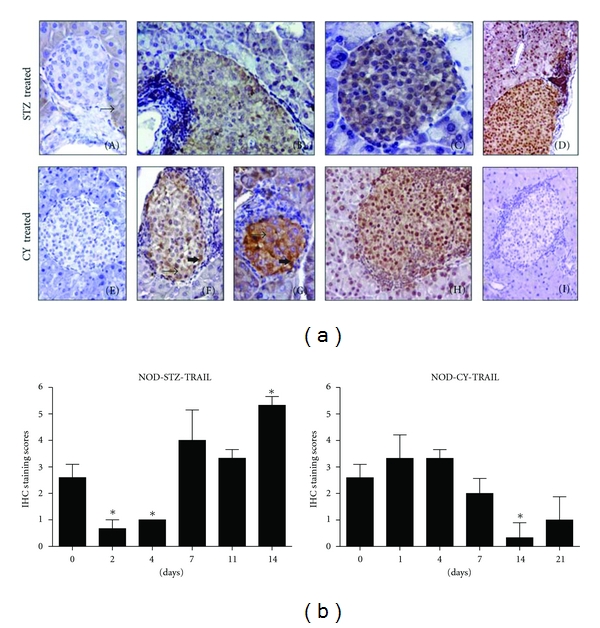
Histologic examination of the pancreatic islets of STZ- or CY-treated NOD mice for TRAIL ligand expression. NOD mice received single intraperitoneal injections of 150 mg/kg STZ or 200 mg/kg CY. Pancreata were isolated at days 0, 2, 4, 7, 11, and 14 in the STZ group, and at days 0, 1, 4, 7, 14, and 21 in the CY group. Three mice were sacrificed at each point. Representative images are shown for negative, weak, moderate, and strong stainings for the TRAIL ligand expression. (a) Sample stainings for STZ-treated mice are shown through (A)–(D), and for CY-treated mice through (E)–(H): (A) and (E) show negative staining for TRAIL in the pancreatic islet in the STZ- and CY-treated groups, respectively; the surrounding acinar cells display weak IC staining (arrow) in (A). Weak IC (B), moderate IC (C), and strong nuclear (D) positivity in the pancreatic islets. Acinar cells display strong nuclear positivity in (D); (F) presents weak (thin arrow) and moderate (thick arrow) IC stainings within the islet, (G) moderate (thin arrow) and strong (thick arrow) IC positivity in the islet, (H) moderate IC and strong nuclear staining in majority of the islet, and moderate nuclear positivity in acinar cells. Control staining (secondary antibody only) is shown in (I). Smaller islets are depicted at 400X magnification for clarity, while other images were taken at 200x. (b) Immunohistochemical staining results for TRAIL were evaluated as described in Section 2. Changes at days 2, 4, and 14 observed in the STZ group, and the alteration observed at day 14 in the CY group were statistically significant (*P* < 0.05). Error bars represent ±SEM. IC: intracytoplasmic.

**Figure 4:**
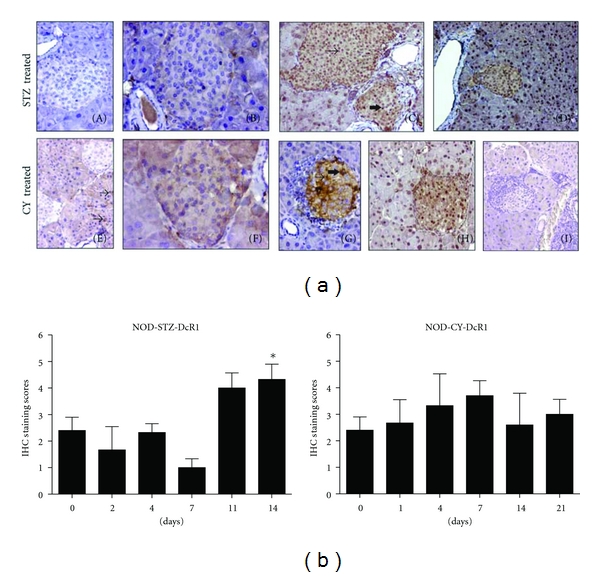
DcR1 expression analysis in STZ- or CY-treated NOD mice. Pancreata were isolated at days 0, 2, 4, 7, 11, and 14 in the 150 mg/kg STZ-injected group and at days 0, 1, 4, 7, 14, and 21 in the 200 mg/kg CY-injected group. Representative images are shown for negative, weak, moderate, and strong stainings for DcR1 expression. (a) Sample stainings for STZ-treated mice are displayed through (A)–(D), and for CY-treated mice through (E)–(H): negative staining for DcR1 is shown in (A) and (E); moderate IC staining is evident in some acinar cells in (E), depicted by arrow. (B) Weak IC positivity in the islet, moderate IC staining in the acinar cells, (C) weak IC, moderate nuclear (thin arrow) and strong nuclear (thick arrow) staining in the islet, moderate nuclear staining in acinar cells, (D) weak IC and strong nuclear positivity in the islet, along with strong nuclear positivity in acinar cells, (F) weak IC staining in the islet, moderate IC positivity in some acinar cells, (G) moderate (thin arrow) and strong (thick arrow) positivity in the islet, no staining in acinar cells, (H) moderate IC, as well as strong nuclear stainings in the islet, moderate nuclear staining in the acinar cells, (I) control staining with secondary antibody alone. Smaller islets are depicted at 400x magnification for clarity. Other images were taken at 200x. (b) Immunohistochemical staining evaluations for DcR1 were performed as described in Section 2. Each bar represents mean data from three mice. Increase at day 14 in the STZ group is significant (*P* < 0.05). Error bars represent ±SEM. IC: intracytoplasmic.

Prior to the analysis of TRAIL ligand and receptors in the pancreatic islets, immunohistochemical stainings were performed on lymph node sections to confirm the specificities of the antibodies used, which revealed strong staining patterns. Incubation of the lymph node with the secondary antibody alone (negative control), in contrast, did not result in any positive staining. Sample lymph node stainings for TRAIL and its DcR1 receptor are shown (Figure 2). Differences in staining intensities between the beta islets and the acinar cells further confirmed specificity (Figures 3 and 4). 

Expression levels of TRAIL ligand and receptors in the pancreatic sections of the subjects were detected by immunohistochemical analysis as described in Section 2. In NOD mice that received STZ as a diabetes-accelerating agent, TRAIL expression displayed a statistically significant yet temporary decrease between the second and fourth days of injection, when at least two out of three mice sacrificed were diabetes free (*P* < 0.05) (Figure 3(b)). However, TRAIL expression levels started to increase in the following days. The rise at day 14 was statistically significant, at a point where all the animals followed were diabetic, compared to expressions detected at day 0 (*P* < 0.05). Data that was acquired from STZ-injected NOR mice (not shown) could not serve as controls for the related data obtained from the NOD mice. This is because NOR mice were very severely affected from STZ induction (see below). 

Following CY application, on the other hand, TRAIL expression was higher during at least the first four days, compared to the basal levels detected prior to injections (Figure 3(b)). All animals were diabetes free at this period. We observed a reduction starting from day 7. At day 14 and 21, TRAIL expression levels were quite lower than the values measured at day 0. The reduction at day 14 was statistically significant (*P* < 0.05). Two out of three animals sacrificed at both points were diabetic. TRAIL expression levels detected in CY-injected NOR mice were generally lower than those of NOD mice acquired at day 0 all throughout the experimental course (data not shown). 

### 3.3. DcR1 Levels Increased in STZ-Applied NOD Mice Islets

**Figure 5:**
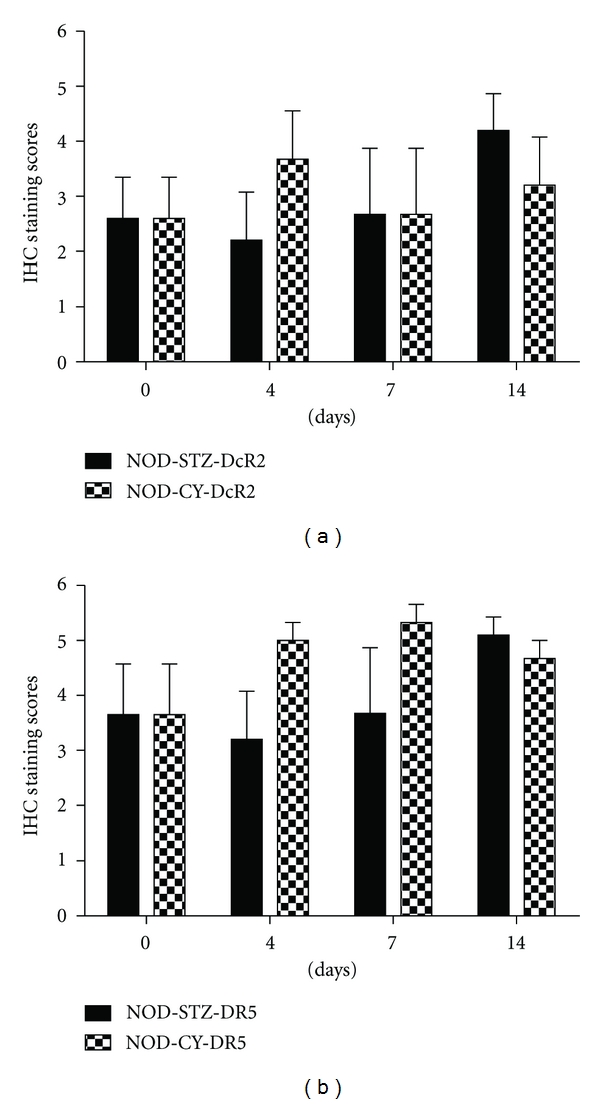
Expression profiles for DcR2 and DR5 in STZ- or CY-applied, NOD mice. Sample collection days for immunohistochemical analyses are given under bars. The collection days shown correspond to the common pancreata collection points in both groups. The mean values for 3 different subjects are presented in each bar. Error bars represent ±SEM. Statistical significance (*) was determined by Mann-Whitney *U *test applied following the Shapiro-Wilk normality test. A *P* value of 0.05 was considered significant.

DcR1 levels increased following STZ injection (Figure 4(b)). Interestingly, DcR1 expression was kept at a relatively low level in the first few days at a mainly diabetes-free stage for all the animals, somewhat similar to what was observed for TRAIL (*P* < 0.05). It reached its highest levels at days 11 and 14, when all the mice were diabetic. The increase recorded at day 14, when all the animals analyzed were diabetic, was statistically significant. No significant change was evident in DcR1 levels after CY application. Although a slight increase in the DR5 and DcR2 receptor levels was evident throughout the development of accelerated diabetes by both agents, the increase was not statistically significant (Figure 5). 

### 3.4. TRAIL Decoy and Death Receptor Expressions Generally Increased in Acinar Cells Following STZ or CY Application, Whereas TRAIL Ligand Expression Levels Were Differentially Affected

**Figure 6:**
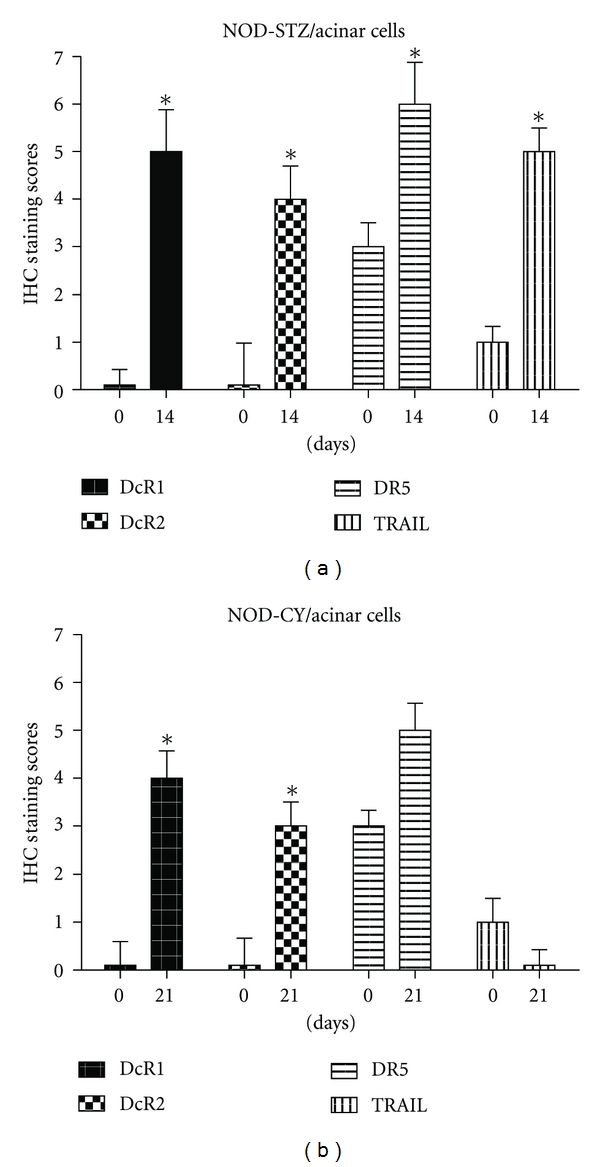
TRAIL ligand and marker expressions in pancreatic acinar cells of STZ- or CY-applied NOD mice. Expression values before agent application (day 0) and at the last pancreas collection points (Day 14 for STZ application and day 21 for CY application) are given. The mean values of 3 different subjects are presented in each bar. Error bars represent ±SEM. Statistical significance (*) was determined by Mann-Whitney *U* test applied following the Shapiro-Wilk normality test. A *P* value of 0.05 was considered significant.

TRAIL receptor expressions increased in the acinar cells of the STZ- and CY-treated NOD mice, compared to the values obtained at day 0, before application of any agents (Figure 6). TRAIL ligand expression, on the other hand, increased by STZ while kept at minimal levels by the action of CY. DcR1 or TRAIL expression in the acinar cells after STZ application did not display a temporary decrease during the first days of application, contrary to what was observed in the islets. While TRAIL ligand expression was already at minimal levels in the acinar cells at day 0, it was kept at minimum all through CY-induced disease. Sample stainings can be viewed in Figures 3 and 4. 

### 3.5. NOR Mice Were Affected More Severely from STZ Compared to the NOD Mice

**Figure 7:**
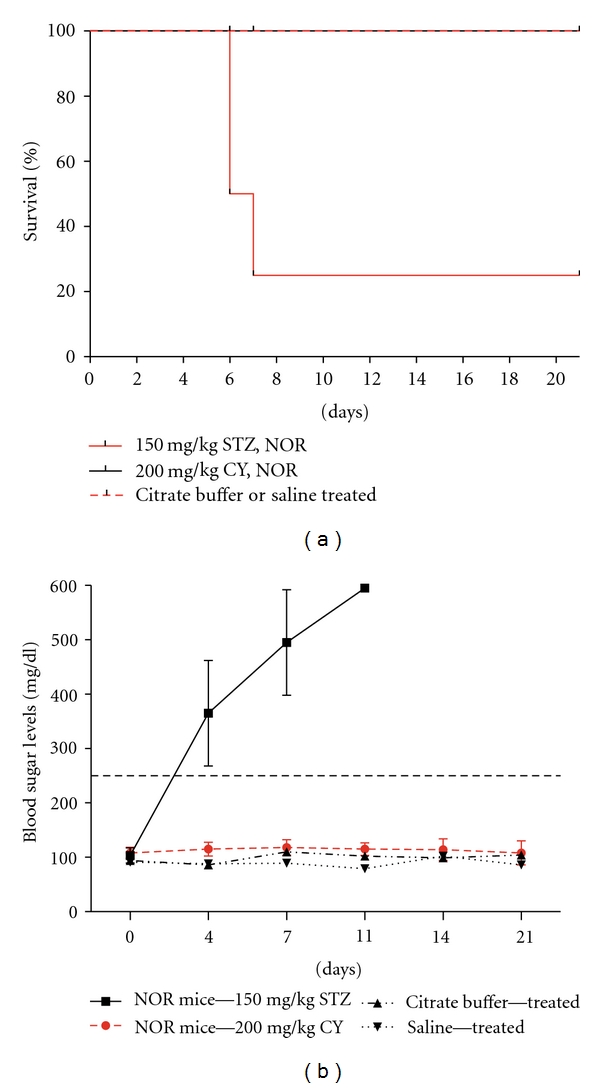
Survival patterns and mean blood glucose level profiles for NOR mice. (a) Mortality rates for NOR mice. Female NOR mice received the same agent doses as the NOD mice, that is, single intraperitoneal injections of 150 mg/kg STZ (*n* = 10), or 200 mg/kg CY (*n* = 10) or vehicle (physiological saline or citrate buffer) at 10 weeks of age. Eighty percent of the STZ-injected mice died at day 7. (b) Changes in blood glucose levels in NOR mice. Nonfasting blood sugar levels of the STZ-, CY-, or vehicle-injected NOR mice groups were measured by a portable glucose meter. Data are given as mean ± SEM.

NOR mice are described as insulitis-resistant and diabetes-free strains and are defined by the producer Jackson Lab as appropriate controls for examining the role of non-MHC genes in development of diabetes. We used 10 NOR mice each for STZ and CY induction groups as controls. They received exactly the same doses as the NOD mice. However, rather unexpectedly, STZ had a more severe effect on the diabetes-resistant NOR mice compared to the diabetes-prone NOD mice. These mice displayed diabetic blood sugar levels (>250 mg/dL) at the second day of STZ application. At day seven, 80% of the mice died (Figure 7). For this reason, NOR mice could not serve as controls for the STZ applications in NOD mice. Instead, expression levels at day 0 of application were used as control values. On the other hand, CY did not display any toxic or diabetic effects on NOR mice. All animals were at good condition all throughout the experimental course following induction, and blood sugar levels stayed under 118 mg/dL at all points (Figure 7). 

## 4. Discussion

Various cytokines such as FasL and TNF-alpha have been related to beta cell destruction in T1D. In contrast, controversial roles were attributed to TRAIL, which is an important component of the immune system. Further studies are required to clarify the exact role of TRAIL in this disease. NOD mice are frequently used in investigations related to T1D, and accelerated disease models provide both a higher prevalence and a faster disease development. STZ and CY are among the most commonly used diabetes-inducing agents for this purpose. We hypothesized that STZ and CY would differentially influence TRAIL ligand and receptor expression levels due to their distinct action mechanisms and that the related findings would provide implications on the role of these marker molecules in T1D development. Our results revealed that TRAIL ligand and DcR1 expression levels were increased by the action of STZ in the pancreatic beta cells of the NOD mice. Whether this may suggest a possible protective role for these two markers for the pancreatic beta cells against the heavily infiltrating lymphocytes may be elucidated by further supportive investigations. CY treatment, on the other hand, led to a definite reduction in the level of TRAIL. Reduction in TRAIL expression in the islets in this instance may contribute to the diabetes-accelerating action of CY. 

We aimed for an intermediate diabetes development rate with the possible highest prevalence when deciding the STZ and CY doses to be applied to test our hypothesis. Varying doses of STZ and CY have been used for acceleration of diabetes in NOD mice [5, 15, 19, 21]. Factors such as the general health condition and age of the subjects at the time of injection, accommodation conditions, route of application, and possibly even slightly differing yet unrecognized genetic backgrounds may all affect the survival of these subjects and diabetes prevalence in induced disease, besides the applied concentration. With regard to STZ treatment, it is well known that a single high dose induces a more rapid loss of insulin secretion and therefore more rapid onset of diabetes in mice. We ran a pilot study with a high dose of the agent at the start of our investigation. A single dose of 200 mg/kg STZ was severely toxic for the NOD mice (*n* = 5) and caused majority of the animals to die at day 6. Yet, no prominent or premature severe toxicity was observed at 150 mg/kg (Figure 1). Although the effect of a single and relatively high dose of STZ on the expression levels of TRAIL ligand and receptors was detected in this study, it may be meaningful to compare this effect with that in a disease setting established with lower and multiple doses of the agent. On the other hand, a single dose of 200 mg/kg CY treatment applied to 10 weeks old female NOD mice provided a suitable model (Figure 1). Consistently, this dose has also been preferred in many other reports [3, 5, 19]. 

In contrast to the increased expressions of TRAIL ligand and DcR1 observed in relatively advanced disease stages in our study (discussed below), a significant reduction was evident in the expression level of especially TRAIL both between days 2 and 4 of STZ injection, while DcR1 levels were also kept low (Figures 3 and 4). Diabetic symptoms (blood sugar levels over 250 mg/kg and gradual weight loss) started at day 4 in the STZ-applied NOD mice group. Activation of the infiltrated T lymphocytes has been reported to be hindered at early stages of diabetes in NOD mice developing spontaneous disease, by the action of an immune regulator mechanism. Beta cells get into a temporary proliferative cycle at this stage, possibly in an effort to compensate for the damage. Following this, a wide-ranged destruction of the beta islets occurs with T-cell activation, most likely due to the ongoing presence of beta cell antigens [22, 23]. Further investigation is required to find out if this mentioned proliferative stage at the start of diabetic symptoms is associated with the low levels of TRAIL and DcR1 expressions detected in our study following STZ injection. 

It is well accepted that the diabetes-inducing agents STZ and CY have different disease-causing mechanisms. In the immunohistochemical analysis performed following STZ induction, a significant increase was detected in the expression levels of the TRAIL ligand and DcR1 receptor expressions in the pancreatic islets of NOD mice at day 14, when all the subjects followed were diabetic, compared to day 0 (prior to STZ injection) (Figures 3 and 4). Expression levels of these markers may have increased in islet cells in response to the rapid development of diabetes following STZ injection accompanied with a heavy lymphocyte infiltration. Increased TRAIL expression has been shown in the cytokine-induced beta islets by Mi et al. [5]. Also, in a recent study by our group, TRAIL overexpression was used as a strategy to increase its expression levels in the allotransplanted beta islets to diabetic rats, thus providing a higher protection against the infiltrating leukocytes. We showed that high TRAIL expression provided in the transplanted pancreatic islets by adenoviral vectors decreased severity of insulitis in STZ-induced diabetic rats, while it prolonged allograft longevity and increased its function [24]. The increase we observed in the level of DcR1 expression may also reflect a defensive strategy. This is because the infiltrating leukocytes are also known to express the TRAIL ligand themselves, which is thought to be one of the apoptotic ligands used by these cells to promote apoptosis in the beta cells. It was shown by many groups before including ours that elevated decoy receptor levels on the cell surface might provide resistance to TRAIL. So, this elevation detected in DcR1 levels on the pancreatic beta cells may be an initial yet eventually inadequate effort to resist the apoptotic effect of TRAIL expressed by the infiltrating lymphocytes. 

DcR2 and DR5 levels were generally expressed at considerable amounts in the islets in STZ-applied, as well as CY-applied NOD mice throughout diabetes development (Figure 7). Although both agents seemed to cause an increase in the expression levels of these markers, the differences in the expression levels were not significant compared to day 0 values. A statistically significant increase in DR5 levels in particular may have been expected, as STZ is known to increase p53 levels, which in turn elevates the DR5 death receptor expression [25, 26]. 

TRAIL ligand expression significantly decreased in CY-injected NOD mice islets (Figure 5). CY is known to be an immune suppressor agent. It is reported to be effective on especially the CD4^+^CD25^+^ regulator T cells (Tregs). Treg cells suppress CD8^+^ T-cell population in normal conditions. However, a high level of apoptosis was reported in Tregs isolated from CY-applied mice, with their ability to suppress CD8^+^ T-cells lost [27]. Brode et al. then detected Tregs as the target of CY, after they specifically identified these cells with the natural Treg marker Foxp3 [19]. The significant decrease in our study in the level of TRAIL may be meaningful in this regard, as TRAIL is known to be an important component of the immune system, and lack of TRAIL leads to a faster T1D development. In support with this, spontaneous diabetes in NOD mice did not lead to a decrease in Treg cells [28]. The same study claimed that spontaneous diabetes development could not be accounted to a qualitative or quantitative decrease in the regulator T cells, but rather to alterations in the frequency and function of the pathogenic T cells. Thus, CY seems to both suppress Treg cells and decrease TRAIL expression in pancreatic islets, both of which can be accounted for accelerated diabetes development. 

The TRAIL ligand and receptor expression profiles obtained in the acinar cells tend to strengthen the specificity of the effects of the two different diabetes-accelerating agents on the islets and somewhat in the acinar cells. As well accepted, STZ is taken up by the beta cells through GLUT2 receptors, which are known as beta-cell-specific glucose transporter molecules. We discussed above that in the pancreatic islets, where beta cells are the most prevalent cell type, we observed significant increases in DcR1 and TRAIL levels after a temporary yet significant decrease between days 2 and 4 of STZ application for TRAIL (Figures 3 and 4) and that expressions of the two other receptors (DcR2 and DR5) were in contrast not altered significantly (Figure 5). Since the GLUT2 receptor expression is specific to beta cells and thus not found in the acinars, the effect of STZ on the acinar cells (gradual increase in TRAIL ligand and receptor expressions throughout the disease course) may have appeared as a result of the stress experienced by these cells in response to a toxic agent introduced to their environment, but not taken up inside, and also by the effect of advanced disease. CY, on the other hand, may have a suppressive effect on TRAIL also, besides the T regulatory cells. This differential effect by CY seems to be confirmed on acinar cells also, where TRAIL expression was kept at minimum throughout the CY-accelerated disease. TRAIL is an important component of the immune system that is thought to be expressed as a protective measure by the pancreatic islets against the infiltrating lymphocytes. While all the TRAIL receptor expressions generally increased in the acinar cells following CY application, with the increase in DcR1 and DcR2 being statistically significant (*P* < 0.05), none of the TRAIL receptor expressions displayed any significant alteration in the beta islets. This seems to support a still differential effect by CY on the islets and the acinar cells. 

Various different mice strains may be utilized as controls in studies on NOD mice, depending on the nature of the study and the related hypothesis. NOR mice are described by the producer Jackson Lab as insulitis-resistant and diabetes-free strains. While reported to still exhibit peripheral T-lymphocyte accumulation and defective peritoneal macrophage responses characteristic of NOD mice, they are defined as useful for examining the role of non-MHC genes in development of diabetes. While STZ application at a dose of 150 mg/kg provided diabetes development in NOD mice starting from day 4 and did not present undesired premature toxicity, the same dose rather unexpectedly caused the death of 80% of the NOR mice at day 7 due to a rapid rise in blood glucose levels which resulted in severe disease manifestation (Figure 7). This severe effect exerted by STZ application on a mice strain known as diabetes resistant may be due to a somewhat higher toxicity of the agent on these mice, presumably through a higher exposition by a yet unknown mechanism. STZ is an antibiotic produced by *Streptomyces achromogenes* and exerts its cytotoxic effect mainly by nitric oxide formation and alkylation of DNA [16]. It is known as a glucose analogue accumulating selectively in pancreatic beta cells via GLUT2 receptor [29]. Possible higher GLUT2 receptor composition in NOR mice may be the underlying mechanism, which is currently investigated by our group. CY, on the other hand, produced no cytotoxic/diabetic effects on NOR mice. This supports the notion that diabetes induction by CY requires a pathological background such as insulitis, which was evident in NOD mice [15]. As NOR mice could not serve as controls for the STZ group, expression levels obtained at day 0 before agent applications were used as basic references for comparing STZ- versus CY-mediated alterations.

Our results also implicate that while T1D models are appropriate for wide use in the related research, differences in the disease-causing mechanisms of different agents should be taken into consideration. Other significant components of the immune system than TRAIL are highly likely to be affected distinctly in response to different agents. Further studies on the subject with wider experimental and control groups and perhaps on alternative animal models as well will contribute to the clearance of the above discussed implications.

## 5. Conclusions

Using NOD mice, we found that TRAIL and DcR1 expression levels are differentially influenced in the pancreatic islets in STZ- versus CY-accelerated T1D. Elevated levels of both markers detected in STZ-accelerated disease in the islets may indicate a defense mechanism against the infiltrating lymphocytes. On the other hand, the contrary reduction in TRAIL expression observed following CY application might be a part of the disease-causing mechanism of CY, in addition to the well-proved suppressive effect of this agent on the T regulatory cells.

## Figures and Tables

**Table 1:** Proportions of diabetic subjects in the sacrificed groups at each point in STZ- or CY-applied NOD mice groups.

STZ group (start of diabetes: day 4)					CY group (start of diabetes: day 11)				
Day 2	Day 4	Day 7	Day 11	Day 14	Day 1	Day 4	Day 7	Day 14	Day 21
0/3	1/3	3/3	3/3	3/3	0/3	0/3	0/3	2/3	2/3
